# Associations between changes in habitual sleep duration and lower self-rated health among COVID-19 survivors: findings from a survey across 16 countries/regions

**DOI:** 10.1186/s12889-023-17258-3

**Published:** 2023-11-28

**Authors:** Kentaro Matsui, Frances Chung, Adrijana Koscec Bjelajac, Ilona Merikanto, Maria Korman, Sérgio Mota-Rolim, Ana Suely Cunha, Bjørn Bjorvatn, Pei Xue, Christian Benedict, Charles M. Morin, Colin A. Espie, Anne-Marie Landtblom, Thomas Penzel, Luigi De Gennaro, Brigitte Holzinger, Harald Hrubos-Strøm, Damien Leger, Courtney J. Bolstad, Michael R. Nadorff, Giuseppe Plazzi, Catia Reis, Ngan Yin Chan, Yun Kwok Wing, Juliana Yordanova, Yves Dauvilliers, Markku Partinen, Yuichi Inoue

**Affiliations:** 1 Department of Clinical Laboratory, National Center Hospital, National Center of Neurology and Psychiatry, Kodaira, Japan; 2 Department of Anesthesiology and Pain Medicine, University Health Network, University of Toronto, Toronto, Ontario Canada; 3 Institute for Medical Research and Occupational Health, Zagreb, Croatia; 4 SleepWell Research Program, Faculty of Medicine, University of Helsinki, Helsinki, Finland; 5 Orton Orthopaedics Hospital, Helsinki, Finland; 6 Department of Occupational Therapy, Faculty of Health Sciences, Ariel University, Ariel, Israel; 7 Brain Institute, Physiology and Behavior Department, and Onofre Lopes University Hospital, Federal University of Rio Grande do Norte, Natal, Brazil; 8 Medical College, Potiguar University, Natal, Brazil; 9 Department of Global Public Health and Primary Care, University of Bergen, Bergen, Norway; 10 Norwegian Competence Center for Sleep Disorders, Haukeland University Hospital, Bergen, Norway; 11 Department of Pharmaceutical Biosciences, Molecular Neuropharmacology, Uppsala University, Uppsala, Sweden; 12 Centre de recherche CERVO/Brain Research Center, École de psychologie, Université Laval, Quebec City Quebec, Canada; 13 Sir Jules Thorn Sleep and Circadian Neuroscience Institute, Nuffield Department of Clinical Neurosciences, University of Oxford, Oxford, UK; 14 Department of Medical Sciences, Neurology, Uppsala University, Uppsala, Sweden; 15 Department of Biomedical and Clinical Sciences, Linköping University, Linköping, Sweden; 16 Sleep Medicine Center, Charite University Hospital Berlin, Berlin, Germany; 17 Department of Psychology, Sapienza University of Rome, Roma, Lazio Italy; 18 IRCCS Fondazione Santa Lucia, Roma, Italy; 19 Institute for Consciousness and Dream Research; Medical University of Vienna, Postgraduate Sleep Coaching, WienVienna, Austria; 20 Department of Otorhinolaryngology, Akershus University Hospital, Lørenskog, Norway; 21 Institute of Clinical Medicine, University of Oslo, Oslo, Norway; 22 VIFASOM (EA 7331 Vigilance Fatigue Sommeil et Santé Publique), Université Paris Cité, Paris, France; 23 APHP, Hôtel-Dieu, Centre du Sommeil et de la Vigilance, Paris, France; 24 Department of Psychology, Mississippi State University, Mississippi State, MS USA; 25 South Texas Veterans Health Care System, San Antonio, Texas USA; 26 IRCCS Istituto Delle Scienze Neurologiche di Bologna, Bologna, Italy; 27 Department of Biomedical, Metabolic and Neural Sciences, University of Modena and Reggio Emilia, Modena, Italy; 28 Católica Research Centre for Psychological - Family and Social Wellbeing, Universidade Católica Portuguesa, Lisbon, Portugal; 29 Instituto de Medicina Molecular João Lobo Antunes, Faculdade de Medicina de Lisboa, Universidade de Lisboa, Lisboa, Portugal; 30 Instituto de Saúde Ambiental, Faculdade de Medicina, Universidade de Lisboa, Lisboa, Portugal; 31 Li Chiu Kong Family Sleep Assessment Unit, Department of Psychiatry, Faculty of Medicine, The Chinese University of Hong Kong, Hong Kong SAR, China; 32 Institute of Neurobiology, Bulgarian Academy of Sciences, Sofia, Bulgaria; 33 Sleep-Wake Disorders Center, Department of Neurology, Gui-de-Chauliac Hospital, Institute for Neurosciences of Montpellier INM, INSERM, University of Montpellier, Montpellier, France; 34 Department of Clinical Neurosciences, University of Helsinki Clinicum Unit, Helsinki, Finland; 35 Helsinki Sleep Clinic, Terveystalo Healthcare Services, Helsinki, Finland; 36 Japan Somnology Center, Institute of Neuropsychiatry, Tokyo, Japan; 37 Department of Somnology, Tokyo Medical University, Tokyo, Japan

**Keywords:** Coronavirus infections, Health status indicators, Sleep wake disorders, Fatigue, Post-Acute Sequelae of SARS-CoV-2 Infection (PASC)

## Abstract

**Background:**

Self-rated health (SRH) is widely recognized as a clinically significant predictor of subsequent mortality risk. Although COVID-19 may impair SRH, this relationship has not been extensively examined. The present study aimed to examine the correlation between habitual sleep duration, changes in sleep duration after infection, and SRH in subjects who have experienced SARS-CoV-2 infection.

**Methods:**

Participants from 16 countries participated in the International COVID Sleep Study-II (ICOSS-II) online survey in 2021. A total of 10,794 of these participants were included in the analysis, including 1,509 COVID-19 individuals (who reported that they had tested positive for COVID-19). SRH was evaluated using a 0-100 linear visual analog scale. Habitual sleep durations of < 6 h and > 9 h were defined as short and long habitual sleep duration, respectively. Changes in habitual sleep duration after infection of ≤ -2 h and ≥ 1 h were defined as decreased or increased, respectively.

**Results:**

Participants with COVID-19 had lower SRH scores than non-infected participants, and those with more severe COVID-19 had a tendency towards even lower SRH scores. In a multivariate regression analysis of participants who had experienced COVID-19, both decreased and increased habitual sleep duration after infection were significantly associated with lower SRH after controlling for sleep quality (*β* = **−**0.056 and **−**0.058, respectively, both *p* < 0.05); however, associations between current short or long habitual sleep duration and SRH were negligible. Multinomial logistic regression analysis showed that decreased habitual sleep duration was significantly related to increased fatigue (odds ratio [OR] = 1.824, *p* < 0.01), shortness of breath (OR = 1.725, *p* < 0.05), diarrhea/nausea/vomiting (OR = 2.636, *p* < 0.01), and hallucinations (OR = 5.091, *p* < 0.05), while increased habitual sleep duration was significantly related to increased fatigue (OR = 1.900, *p* < 0.01).

**Conclusions:**

Changes in habitual sleep duration following SARS-CoV-2 infection were associated with lower SRH. Decreased or increased habitual sleep duration might have a bidirectional relation with post-COVID-19 symptoms. Further research is needed to better understand the mechanisms underlying these relationships for in order to improve SRH in individuals with COVID-19.

**Supplementary Information:**

The online version contains supplementary material available at 10.1186/s12889-023-17258-3.

## Background

Self-rated health (SRH) is a single-term indicator of perceived health and is relatively reliable in predicting one’s morbidity and mortality [1, 2]. SRH is interpreted as a subjective summary of an individual's health status that takes into account biological, mental, social, and functional aspects of a person [3], but is reportedly also correlated with biomarkers representative of various organ systems [4]. The association between SRH and mortality becomes weaker when comorbidities and functional status are statistically adjusted, but it remains as a marker predicting a nearly two-fold increase in mortality risk that cannot be explained by objective health measures [5]. Recently, various physical symptoms that persist after SARS-CoV-2 infection, described as post-COVID-19 conditions or *'*Long COVID,*'* have become a significant concern [6]. Possibly due to this, there are some reports about poor SRH in individuals affected with COVID-19 [7, 8].

Generally, acute infectious diseases are likely to cause various physiological and behavioral responses, collectively known as "sickness behaviors," through cytokine responses [9], which also affect sleep [10]. Similarly, in COVID-19, in addition to a high rate of subjective sleep disturbance [11, 12], some individuals experience changes in their habitual sleep duration toward either decreasing or increasing direction relative to that before the infection [13]. To date, no study has examined the relationship between changes in habitual sleep duration following COVID-19 and subsequent health outcomes. Although previous studies have shown that both short and long sleep durations are generally associated with lower SRH [14–21], it is unclear whether this association holds true for individuals who experienced COVID-19.

The International COVID Sleep Study (ICOSS) was started as an international collaborative research project involving sleep experts from Europe, Asia, North America, and South America during the COVID-19 pandemic in 2020 [22]. The second survey (ICOSS-II) was conducted in 2021 to investigate the interaction among sleep, the severity of infection, and health issues in the general population during the pandemic. In the ICOSS-II, data on SRH are available for both non-infected and infected individuals [23]. The objective of the present study was to determine, in participants who experienced COVID-19, 1) whether those with habitual short or long sleep duration had worse SRH compared to those with normal sleep duration, 2) whether a greater change in habitual sleep duration after the infection was associated with lower SRH, and 3) which post-COVID-19 symptoms were related to the changes in habitual sleep duration before and after the infection. We also investigated the relation of SRH to demographic, social, and COVID-19 variables including the severity of the infection and post-COVID-19 symptoms, as well as to their overall sleep quality.

## Methods

### Setting and study population

Data collection took place in Austria, Brazil, Bulgaria, Canada, China (Hong Kong), Croatia, Finland, France, Germany, Israel, Italy, Japan, Norway, Portugal, Sweden, and the United States from May to December 2021. The survey was mainly conducted using an online platform (Qualtrics and RedCap) and the survey questionnaire was translated into the major languages of each region. Participants were recruited through university websites, local and national newspapers, professional societies, television, Facebook, and Twitter in each country [23]. All respondents were anonymous volunteers with age over 18 years old. A total of 15,813 people participated in the ICOSS-II, but 5,131 were excluded from the analysis mainly because they did not respond to SRH, as detailed in Supplementary Table 1. The final sample consisted of 10,663 participants.

### Main outcome measures

SRH was assessed using a 0–100 linear visual analog scale (VAS). In the questionnaire, participants were asked: "Draw a line on the ruler below that best fits your current quality of health at the moment (past three months): 0 means worst possible health (almost dead) and 100 indicates totally healthy, extremely healthy.” This scale has 0 indicating the worst possible health and 100 representing the best possible SRH [3, 24].

### Candidate predictive variables—COVID-19 status

All participants were asked to self-report the severity of infection if they had tested positive for COVID-19 (no; yes). For COVID-19 affected individuals, disease severity was assessed using the following five response options: no marked symptoms; mild symptoms (symptoms disappeared without taking specific medications); moderate symptom (medications were used, but caused no severe pneumonia, no extra oxygen inhalation was needed); severe symptom (received extra-oxygen inhalation due to pneumonia); and life-threatening symptom (required invasive ventilation or maximum available respiratory support).

Based on the WHO criteria [6], the post-COVID-19-symptoms were assessed if the participant had suffered for ≥ 3 months after the initial SARS-CoV-2 infection for the following 19 symptoms: a) shortness of breath/difficulty breathing/chest pain , b) joint pain (arthralgia)/muscle pain, muscle aches, c) migraine, d) headache other than migraine, e) abdominal pain, colic, f) palpitations/cardiac arrhythmia, g) tachycardia, fast pulse rate, h) post-exertional malaise referring to prolonged weakness/poor functionality after exertion, such as muscle weakness, difficulties walking long distances, i) dizziness when standing, j) low blood pressure (hypotension), k) urinary problems, l) problems of sweating/trouble tolerating cold/heat, m) problems of attention or concentration/brain fog, cognitive dysfunction, memory problems, n) loss of smell/taste, o) hallucinations, psychotic symptoms, p) feverishness/flu-like symptoms, such as sore throat, runny nose, etc., q) diarrhea/nausea/vomiting, r) symptoms of fatigue, and s) excessive daytime sleepiness. In this study, the total number of symptoms meeting the above 19 post-COVID-19 items was counted in participants who tested positive for COVID-19.

### Candidate predictive variables —habitual sleep duration and its change

In this survey, the response to the question "How many hours per night have you been sleeping on average currently (hours, minutes)?" was used as habitual self-reported nighttime sleep duration. This was then divided into three categories: short sleep duration (<6 h/night); reference sleep duration (6 to 9 h/night); and long sleep duration (>9 h/night). These cutoff points were chosen because they have been considered to represent problematic short or long sleep durations [18, 25–27]. The participants who tested positive for COVID-19 were asked about their average sleep duration before infection with the question "How many hours per night did you sleep on average during the pandemic, before having COVID-19 (hours, minutes)?" A significant number of participants reported no change in sleep duration when comparing values from before the infection to the current state, leading to a skewed distribution. Furthermore, as reported in [28], the general population experienced a reduction in habitual sleep duration as the pandemic progressed. To address this, our analysis employed octiles to segment the data on changes in habitual sleep duration. Consequently, we formed three distinct groups: the first 12.5% of participants who experienced a reduction in sleep duration of ≤ −2 h; the uppermost 12.5% who observed an increase in sleep duration of ≥ 1h; and the remaining central 75% who had sleep duration variations ranging between > −2 h and < 1 h (reference).

### Other independent variables

The following participant characteristics were defined as potential confounders: age, gender, body mass index (BMI), ethnicity (binary: Caucasian/white or others), marital status (binary: married/in relationship or single/divorced/separated/widowed), education (binary: those with vs. without a degree from the university, college or above), number of pre-existing comorbidities (including hypertension, heart failure, stroke, diabetes, asthma, chronic obstructive pulmonary disease, kidney failure, cancer, autoimmune disease, problems of movement and migraine), number of COVID-19 vaccine doses (divided into three categories: never, once or twice), financial burden (divided into three categories: not at all, a little/somewhat, or much/very much) [23]. In addition, given that individuals' own sleep quality may influence their subjective sleep duration [29], we assessed overall sleep quality using a 5-point scale (well, rather well, neither well nor badly, rather badly, or badly), which was assessed in response to the question, "How well have you been sleeping currently? [23]"

### Statistical analysis

The participants’ characteristics were summarized using mean (standard deviation [SD]) scores or percentages. Independent sample t-tests or chi-square test were conducted to investigate potential differences in sociodemographic variables between participants with COVID-19 and those without COVID-19. A one-way analysis of variance Bonferroni's multiple-comparison test was used to compare the differences in SRH by severity of COVID-19 symptoms, current sleep duration, and change in habitual sleep duration after infection. Multiple linear regression analysis was used to examine the association between independent variables and the levels of SRH. For the non-infected group, a multivariable linear regression analysis was conducted targeting the associations of the following covariates: current habitual sleep duration (< 6 h, 6 to 9 h [reference], or > 9 h), age, gender, BMI, ethnicity, marital status, education, number of pre-existing comorbidities, number of COVID-19 vaccine doses, financial burden, and overall sleep quality to the levels of SRH. For the COVID-19 group, the analysis was conducted with the following covariates: current habitual sleep duration and the change in the duration, age, gender, BMI, ethnicity, marital status, education, number of pre-existing comorbidities, number of COVID-19 vaccine doses, financial burden, severity of COVID-19, number of post-COVID-19 symptoms, and overall sleep quality. Taking into consideration that appropriate sleep duration varies with age [30], we conducted a sensitivity analysis using the National Sleep Foundation's recommendations as the cutoff duration [31]. Specifically, we defined current short habitual sleep duration as less than 6 hours for individuals 64 years and younger and less than 5 hours for older adults. Additionally, current long habitual sleep duration was defined as more than 11 hours for individuals 25 years and younger, more than 10 hours for individuals between 26 and 64 years of age, and more than 9 hours for individuals 65 years and older. Furthermore, due to potential confounding effects from differences in participants' countries, we conducted a sensitivity analysis adjusting for country fixed effects. This analysis showed results using multiple regression, where 15 of the 16 studied nations were incorporated as dummy variables. Lastly, a multinomial regression analysis was computed between change in habitual sleep duration (≤ -2 h, > -2 and < 1 h [reference], and ≥ 1h) and each of the 19 post-COVID-19 symptoms, to clarify which of the post-COVID-19 symptoms investigated in the present study had associations with the reduction or extension of habitual sleep duration after infection. However, daytime sleepiness was excluded from the analysis because it can be directly related to changes in sleep duration. The multiple linear regression and multinomial regression were weighted by giving an equal share for each country, and by the joint distribution of age and gender of each participating country to enhance the representativeness of each country's population. However, in the sensitivity analysis examining the fixed effects of countries, we did not apply weighting due to concerns about over-controlling for country differences. We used SPSS statistics version 22 (SPSS Japan, Inc., Tokyo Japan) to perform all analyses. Statistical significance was set at *p* < 0.05.

## Results

The 10,794 participants had a mean age of 43.0 (SD: 16.8) years and approximately two-thirds were female. Responses were received from various countries/regions: Austria (525), Brazil (192), Bulgaria (280), Canada (450), Croatia (513), Finland (1,091), France (303), Germany (502), Hong Kong (394), Israel (354), Italy (824), Japan (2,935), Norway (567), Portugal (430), Sweden (669), and the USA (765). A total of 1,509 participants (14.0%) reported that they had tested positive for COVID-19. These participants were younger, had a higher proportion of woman, a higher BMI, a larger proportion of Caucasians/white people, a lower level of education, a greater number of pre-existing comorbidities, a smaller number of COVID-19 vaccine doses, had suffered more economic hardship due to the pandemic, had a more disrupted sleep quality, and a lower SRH compared to non-infected individuals. There was no significant difference in current habitual sleep duration between the infected and non-infected participants. Approximately two-thirds of infected participants had no marked symptoms or mild symptoms, but 8.5% of them had severe or life-threatening condition. Furthermore, 63.2% of infected participants had at least one long-lasting post-COVID-19 symptom and the mean number of post-COVID-19 symptoms was 3.7 (SD: 4.2). Compared to the habitual sleep duration before infection, the current habitual sleep duration was decreased by approximately 30 minutes in the infected group (Table 1).

**Table 1:** Characteristics and clinical variables of the study participants

	Total (*n* = 10,794)	COVID-19 group (*n* = 1,509)	Not reported positive group (*n* = 9,285)	*p*
Age, mean (SD), years	43.0 (16.8)	40.1 (15.4)	43.5 (17.0)	< 0.001
Gender, %				
Male	32.8	25.2	34.1	< 0.001
Female	67.2	74.8	65.9	
Body mass index, mean (SD), kg/m^2^	24.9 (6.5)	26.5 (8.2)	24.7 (6.1)	< 0.001
Ethnicity, %				
Caucasian/white	61.8	83.8	58.2	< 0.001
Others (Asian, African, Hispanic and others)	38.2	16.2	41.8	
Marital status, %				
Single/divorced/separated/widowed	41.9	40.8	42.1	0.342
Married/in relationship	58.1	59.2	57.9	
Education, %				
University, college or above	64.6	68.7	63.9	< 0.001
Others	35.4	31.3	36.1	
Number of comorbidities, mean (SD)	0.5 (0.8)	0.7 (1.0)	0.5 (0.8)	< 0.001
Number of COVID-19 vaccine doses, %				
Never	22.0	33.2	20.2	< 0.001
Once	14.8	30.2	12.3	
Twice	63.2	36.6	67.5	
Financial burden, %				
Not at all	62.3	58.9	62.8	< 0.001
A little/somewhat	29.6	30.1	29.6	
Much/very much	8.1	11.0	7.6	
Severity of COVID-19, %				
No marked symptoms	—	10.5	—	—
Mild	—	56.6	—	
Moderate	—	24.4	—	
Severe	—	6.8	—	
Life-threatening	—	1.7	—	
Number of post-COIVD-19 symptoms, mean (SD)	—	3.7 (4.2)	—	—
Habitual sleep duration before infection, mean (SD), min	—	444.9 (81.3)	—	—
Current habitual sleep duration, mean (SD), min	416.8 (77.3)	418.2 (82.6)	416.6 (76.5)	0.467
Change in habitual sleep duration, mean (SD), min	—	-26.7 (92.9)	—	—
Sleep quality, %				
Well	19.7	18.8	19.9	< 0.001
Rather well	28.8	26.4	29.2	
Neither well nor badly	26.5	22.5	27.1	
Rather badly	19.1	24.1	18.2	
Badly	5.9	8.2	5.5	
Self-rated health, mean (SD), points	71.8 (20.7)	67.2 (22.6)	72.6 (20.3)	< 0.001

*SD* Standard deviation

Analyses in this table were not weighted

**Fig 1.**
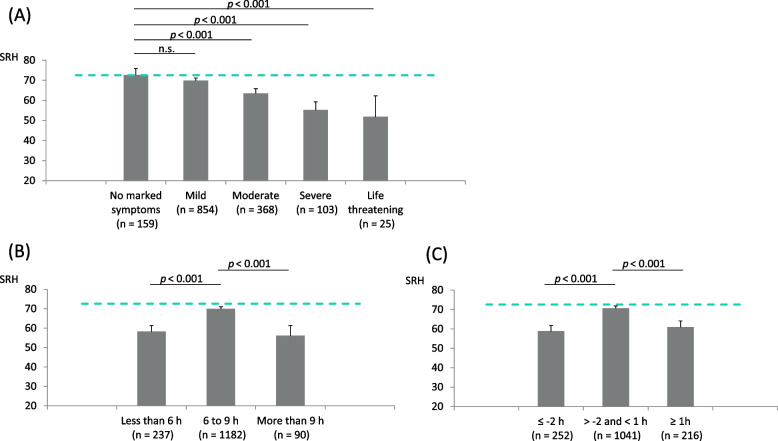
Comparisons of self-rated health according to respective measure categories in the COVID-19 group. Mean self-rated health (SRH) score is shown for respective categories: according to the severity of COVID-19 (**A**), according to current sleep duration (**B**), and according to the change in habitual sleep duration after SARS-CoV-2 infection (**C**). Error bars represent 95% confidence intervals. The dashed green line indicates the mean SRH of the non-infected participants. P-values were calculated through one-way analysis of variance without weighting, followed by Bonferroni post hoc tests

**Fig 2.**
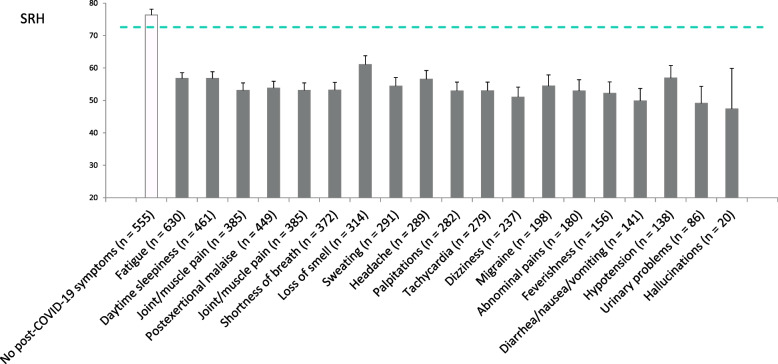
Self-rated health by positivities for respective post-COVID-19 symptoms. Error bars represent 95% confidence intervals. The dashed green line indicates the mean SRH of the participants who did not test positive for COVID-19. Note that the presence of multiple post-COVID-19 symptoms in many individuals of the COVID-19 group precludes the use of a one-way analysis of variance, as it cannot adequately address the resulting interdependencies and complexities

The mean SRH of participants who experienced SARS-CoV-2 infection with different severity (A), their current habitual sleep duration (B), and changes in habitual sleep duration (C) are shown in Fig 1. A significant relation toward a lower SRH was observed with higher severity of COVID-19 (*p* < 0.001). Both short and long current sleep durations showed a significant effect towards a lower SRH (*p* < 0.001). Similarly, both decreased or increased habitual sleep duration after the infection showed a significant effect towards a lower SRH (*p* < 0.001). The mean SRH values for those without post-COVID-19 symptoms and those with respective post-COVID-19 symptoms are shown in Fig. 2.

Table 2 shows the results of the linear regression analyses on factors associated with SRH among participants who did not test positive. Worse SRH was significantly associated with current habitual long sleep duration (> 9 h, *p* < 0.001), higher BMI (*p* < 0.001), non-Caucasian/white (*p* < 0.001), not married/in relationship (*p* < 0.001), more pre-existing comorbidities (*p* < 0.001), fewer number of COVID-19 vaccine doses (*p* < 0.001), higher financial burden (*p* = 0.006), and worse sleep quality (*p* < 0.001), with R-squared of 0.170 (Table 2). The sensitivity analysis using the National Sleep Foundation's cut-offs revealed consistent results, with R-squared of 0.171 (Supplementary Table 2). Another sensitivity analysis that included country fixed effects showed similar results. However, the associations between ethnicity, number of COVID-19 vaccine doses, and SRH were not significant. Conversely, habitual short sleep duration (< 6 h, *p* = 0.015) was independently associated with a decline in SRH after adjusting for country differences. The R-squared value was 0.202 (Supplementary Table 3).

**Table 2:** Multiple linear regression for self-rated health in participants who did not test positive for COVID-19 (*n* = 9,285)

	B	Std. Error	*β*	*p*
Current sleep duration				
< 6 h	−0.457	0.589	−0.008	0.438
6 to 9 h	(ref)			
> 9 h	**−6.089**	**0.973**	**−0.062**	**< 0.001**
Age	−0.006	0.012	−0.005	0.637
Gender: female (yes)	−0.313	0.389	−0.008	0.421
Body mass index	**−0.332**	**0.032**	**−0.105**	**< 0.001**
Ethnicity: Caucasian/white (yes)	**3.566**	**0.427**	**0.084**	**< 0.001**
Marital status: married/in relationship (yes)	**2.242**	**0.408**	**0.057**	**< 0.001**
Education: university, college or above (yes)	−0.492	0.415	−0.012	0.235
Number of comorbidities^a^	**−2.300**	**0.235**	**−0.105**	**< 0.001**
Number of COVID-19 vaccine doses^b^	**0.920**	**0.266**	**0.035**	**< 0.001**
Financial burden^c^	**−2.075**	**0.322**	**−0.066**	**< 0.001**
Sleep quality^d^	**−5.377**	**0.180**	**−0.321**	**< 0.001**

^a^Including hypertension, heart failure, stroke, diabetes, asthma, COPD, kidney failure, cancer, autoimmune disease, problems of movement and migraine

^b^Never=1, Once=2, Twice=3

^c^Not at all=1, A little/Somewhat=2, Much/Very much=3

^d^Well=1, rather well=2, neither well nor badly=3, rather badly=4, badly=5

Analyses weighted by country representation and the joint age-gender distribution

Table 3 shows the results of the linear regression analyses for factors associated with SRH among participants who had experienced COVID-19. After contracting COVID-19, 252 participants (16.7%) experienced a reduction of 2 or more hours in their habitual sleep duration, and 216 participants (14.3%) experienced an extension of 1 or more hours. Worse SRH was significantly associated with decreased (*p* = 0.023) and increased (*p* = 0.016) habitual sleep duration, higher BMI (*p* < 0.001), non-Caucasian/white (*p* = 0.019), larger number of pre-existing comorbidities (*p* = 0.004), higher severity of COVID-19 (*p* < 0.001), larger number of post-COVID-19 symptoms (*p* < 0.001), and worse sleep quality (*p* < 0.001), with R-squared of 0.314 (Table 3). The results of sensitivity analysis, using the National Sleep Foundation's cut-offs, were consistent with those of the main analysis, with R-squared of 0.313 (Supplementary Table 4). Another sensitivity analysis that included country fixed effects showed similar results. However, the association between ethnicity and SRH was not significant. Conversely, habitual long sleep duration (> 9 h, *p* = 0.021), younger age (*p* < 0.001) and not being married or in a relationship (*p* = 0.033) were independently associated with a decline in SRH after adjusting for country differences. The R-squared value was 0.354 (Supplementary Table 5).

**Table 3:** Multiple linear regression for self-rated health in COVID-19 group (*n* = 1,509)

	B	Std. Error	*β*	*p*
Current habitual sleep duration				
< 6 h	0.005	1.539	0.000	0.998
6 to 9 h	(ref)			
> 9 h	−4.436	2.328	−0.044	0.057
Change in habitual sleep duration				
≤ −2 h	**−3.354**	**1.476**	**−0.056**	**0.023**
−2 to 1 h	(ref)			
≥ 1 h	**−3.792**	**1.572**	**−0.058**	**0.016**
Age	0.013	0.032	0.011	0.679
Gender: female (yes)	1.520	0.974	0.035	0.119
Body mass index	**−0.385**	**0.062**	**−0.140**	**< 0.001**
Ethnicity: Caucasian/white (yes)	**2.779**	**1.186**	**0.052**	**0.019**
Marital status: married/in relationship (yes)	1.842	1.056	0.041	0.081
Education: university, college or above (yes)	0.150	1.037	0.003	0.885
Number of comobidities^a^	**−1.608**	**0.560**	**−0.073**	**0.004**
Number of COVID-19 vaccine doses^b^	0.865	0.588	0.032	0.141
Financial burden^c^	−0.686	0.727	−0.021	0.345
Severity of COVID-19^d^	**−2.016**	**0.587**	**−0.080**	**< 0.001**
Number of post-COVID-19 symptoms^e^	**−1.964**	**0.152**	**−0.341**	**< 0.001**
Sleep quality^f^	**−3.342**	**0.455**	**−0.188**	**< 0.001**

^a^Including hypertension, heart failure, stroke, diabetes, asthma, COPD, kidney failure, cancer, autoimmune disease, problems of movement and migraine

^b^Never=1, Once=2, Twice=3

^c^Not at all=1, A little/Somewhat=2, Much/Very much=3

^d^No marked symptoms =1, Mild=2, Moderate=3, Severe=4, Life threatening=5

^e^Including fatigue, brain fog/memory problems, postexertional malaise, joint/muscle pain, shortness of breath, loss of smell, sweating, headache, palpitations, tachycardia, dizziness, migraine, abnominal pains, feverishness, diarrhea, hypotension, urinary problems, and hallucinations

^f^Well=1, rather well=2, neither well nor badly=3, rather badly=4, badly=5

Analyses weighted by country representation and the joint age-gender distribution

Table 4 shows the results of multinomial regression between change in habitual sleep duration and post-COVID-19 symptoms. All the post-COVID-19 symptoms other than loss of smell were significantly associated with either decreased or increased habitual sleep duration in the univariate model. The multiple multinomial regression analysis revealed that prolonged fatigue (odds ratio [OR] = 1.824, 95% CI: 1.193–2.789, *p* = 0.006), shortness of breath (OR = 1.725, 95% CI: 1.128–2.636, *p =* 0.012), dizziness (OR = 0.571, 95% CI: 0.338–0.966, *p =* 0.037), diarrhea/nausea/vomiting (OR = 2.636, 95% CI: 1.417–4.903, *p =* 0.002), and hallucinations (OR = 5.091, 95% CI: 1.263–20.52, *p =* 0.022) were significantly associated with decreased habitual sleep duration; prolonged fatigue (OR = 1.900, 95% CI: 1.214–2.974, *p =* 0.005) was significantly associated with increased habitual sleep duration.

**Table 4:** Multinomial regression analysis on the association between the change in habitual sleep duration and respective post-COVID-19 symptoms (*n* = 1,509)

		Reduction in habitual sleep duration ≥ 2 h, OR (95% CI)		Moderate change in habitual sleep duration (reference)	Extension in habitual sleep duration ≥ 1h, OR (95% CI)	
	n	Univarate	Multivariate		Univarate	Multivariate
Fatigue	630	2.745 (2.064–3.650) ***	1.824 (1.193–2.789) **	ref	2.607 (1.911–3.556) ***	1.900 (1.214–2.974) **
Brain fog/memory problems	557	2.286 (1.714–3.050) ***	1.375 (0.922–2.050) ns	ref	2.142 (1.564–2.934) ***	1.231 (0.799–1.897) ns
Postexertional malaise	449	2.458 (1.824–3.313) ***	0.944 (0.594–1.499) ns	ref	2.089 (1.502–2.906) ***	1.016 (0.616–1.677) ns
Joint/muscle pain	385	2.188 (1.614–2.966) ***	1.178 (0.775–1.792) ns	ref	1.582 (1.117–2.238) **	0.790 (0.496–1.256) ns
Shortness of breath	372	2.697 (1.976–3.681) ***	1.725 (1.128–2.636) *	ref	2.403 (1.705–3.386) ***	1.415 (0.893–2.240) ns
Loss of smell	314	0.973 (0.673–1.406) ns	0.667 (0.438–1.015) ns	ref	1.322 (0.908–1.925) ns	0.908 (0.595–1.386) ns
Sweating	291	2.479 (1.770–3.470) ***	1.350 (0.868–2.099) ns	ref	2.144 (1.474–3.119) ***	1.329 (0.823–2.149) ns
Headache	289	1.580 (1.077–2.318) *	0.635 (0.382–1.055) ns	ref	2.214 (1.508–3.251) ***	1.459 (0.887–2.402) ns
Palpitations	282	2.356 (1.674–3.317) ***	1.027 (0.587–1.794) ns	ref	1.522 (1.012–2.288) *	1.036 (0.556–1.931) ns
Tachycardia	279	2.445 (1.733–3.449) ***	1.271 (0.736–2.195) ns	ref	1.596 (1.059–2.405) *	0.876 (0.470–1.633) ns
Dizziness	237	1.793 (1.215–2.646) **	0.571 (0.338–0.966) *	ref	1.975 (1.308–2.983) **	0.963 (0.560–1.656) ns
Migraine	198	2.263 (1.491–3.433) ***	1.415 (0.823–2.433) ns	ref	1.566 (0.947–2.591) ns	0.828 (0.449–1.527) ns
Abdominal pain	180	1.791 (1.161–2.761) **	0.661 (0.375–1.166) ns	ref	1.100 (0.635–1.906) ns	0.562 (0.292–1.082) ns
Feverishness	156	1.249 (0.748–2.083) ns	0.559 (0.296–1.054) ns	ref	2.102 (1.307–3.381) **	1.124 (0.625–2.022) ns
Diarrhea/nausea/vomiting	141	4.007 (2.535–6.334) ***	2.636 (1.417–4.903) **	ref	2.240 (1.266–3.966) **	1.596 (0.783–3.256) ns
Hypotension	138	2.461 (1.554–3.898) ***	1.419 (0.810–2.484) ns	ref	1.424 (0.792–2.560) ns	0.981 (0.505–1.908) ns
Urinary problems	86	2.589 (1.585–4.229) ***	1.485 (0.831–2.655) ns	ref	1.258 (0.645–2.454) ns	0.846 (0.399–1.795) ns
Hallucinations	20	6.270 (1.832–21.457) **	5.091 (1.263–20.529) *	ref	4.535 (1.124–18.291) *	2.936 (0.596–14.466) ns

*OR* Odds ratio, *Cl* Confidence interval, *ns* Not significant, *ref* Reference

^***^ *p* < 0.001, ** *p* < 0.01, and * *p* < 0.05

Analyses weighted by country representation and the joint age-gender distribution

## Discussion

The present study is the first to study the associations between changes in sleep duration after SARS-CoV-2 infection and the current self-rated health in individuals infected with the coronavirus. The results of this study provided important findings about how the severity of COVID-19 and post-COVID-19 symptoms were related to SRH.

In individuals with COVID-19, the SRH score was lower than that among participants who did not test positive for COVID-19. We found that there was a significant and independent association between the severity of COVID-19 and lower SRH; the infection per se could contribute to the decrease in SRH. Although the relationship between the severity of the infection and subsequent physical outcomes is still controversial [32–36], the association might become more pronounced when evaluated using a subjective measure like SRH [1]. We also found that a higher number of post-COVID-19 symptoms was associated with lower SRH, suggesting that the combination of the post-COVID-19 symptoms may have a greater impact on lowered SRH.

A number of studies have examined the SRH of the general population during the COVID-19 pandemic, and some have raised the possibility of concurrent effects of demographic variables, comorbid physical illnesses, economic conditions, and insomnia of the study population on SRH [37–42]. However, no study under the COVID-19 pandemic has used SRH as the primary outcome variable. In the present study we demonstrate that lower SRH is associated with higher BMI, an ethnic group other than Caucasian, pre-existing comorbidities, and worse overall sleep quality in both the COVID-19 group and the not reported positive group. These findings are largely consistent with those of previous literature, including studies conducted prior to the COVID-19 pandemic [4, 19, 43–51]. The association between ethnicity and SRH was not significant when country fixed effects were included; these associations may be explained by differences in the countries in which respondents resided.

Consistent with a report from Korea [52], the relationship between financial problems due to the pandemic and lower SRH was significant among participants who did not test positive for COVID-19. Economic issues have been one of the significant challenges during the COVID-19 pandemic [53], and they, alongside lockdowns [54, 55], may reflect a strong association with health. Unexpectedly, among participants who did not test positive for COVID-19, the number of vaccine doses received seemed to be protective against lower SRH. Vaccine hesitancy is related to various factors such as individual beliefs, social, cultural, economic, organizational, historical, and political factors [56], and some might have backgrounds with pre-existing comorbidities that could prevent them from receiving vaccination. All these may concurrently contribute to a decline in SRH.

In participants who did not test positive for COVID-19, longer habitual sleep duration was significantly associated with lower SRH. Depression and/or lack of challenge in habitual long sleepers, albeit not controlled in this study [57], may have contributed to the decrease in SRH. Meanwhile, in COVID-19 group, we did not find a significant association between the current short or long habitual sleep duration and lower SRH. This phenomenon might be due to confounding factors related to insomnia symptoms [19, 58, 59] which are represented by the overall sleep quality in this study. By contrast, notably, we found that changes in habitual sleep duration following COVID-19, either decreased or increased, were associated with lower SRH after adjusting for factors such as pre-existing comorbidities and overall sleep quality. Changes in habitual sleep duration that have been observed after SARS-CoV-2 infection [13] may serve as a marker for physical illness; however, no research has been conducted to investigate this correlation. In general, infections can cause either increased or decreased sleep duration [60], and this trend can be applied to COVID-19 [13].

Changes in habitual sleep duration may be influenced by persistent systemic cytokine response [61], and may reflect virus brain penetration and/or the nitric oxide-induced deleterious mechanisms of SARS-CoV-2 [62]. Among the post-COVID-19 symptoms, fatigue was associated with increased habitual sleep duration after infection. The prolonged fatigue, together with abnormal immune responses may be similar to those observed during the development of idiopathic hypersomnia [21, 63], which has been suggested to be triggered by Epstein-Barr infection [64, 65]. Although the evidence is insufficient, SARS-CoV-2 infection itself and/or Epstein-Barr virus reactivation due to SARS-CoV-2 infection may trigger the onset of central nervous system hypersomnias [66, 67]. Meanwhile, shortness of breath and diarrhea/nausea/vomiting might reduce individuals’ habitual sleep duration and lower SRH through their bodily effect. Of note, fatigue, considered as the most common symptom among the post-COVID-19 symptoms [68], was associated with both decreased and increased habitual sleep duration. Reportedly, both short and long sleep duration were associated with the severity of chronic fatigue syndrome/myalgic encephalomyelitis (CFS/ME) [69]. Indeed, post-COVID-19 sequelae resemble the features of CFS/ME [70, 71], suggesting a pathophysiological relationship between post-COVID-19 symptoms and CFS/ME [72], which may partially explain the mechanism behind the lower SRH in COVID-19 individuals. Taken together, these results suggest that a decrease in habitual sleep duration is primarily related to physical sequelae, while an increase in habitual sleep duration may reflect central nervous system fatigue [73]; still, causal relations should be tested in future studies. It is also desirable in future research to verify the effectiveness of interventions aiming at standardizing sleep duration, with the goals of improving SRH and alleviating post-COVID-19 symptoms.

This study has several limitations. First, considering that 20–30% of non-infected individuals experience major post-COVID-19 symptoms [74], the post-COVID-19 symptoms in some participants might have been present even before the infection. In addition, this study did not examine changes in habitual sleep duration in participants who were not infected. Second, this study was a cross-sectional investigation on the relationship between SRH and the current habitual sleep duration or changes in habitual sleep duration following SARS-CoV-2 infection, so the causal nature of the relationship remains unclear. Thus, changes in habitual sleep duration after SARS-CoV-2 infection may be more likely to occur in those who already had low SRH before the infection. Third, previous research on SRH often used Likert scales, while we used VAS. However, VAS possibly has an advantage that it is more sensitive as a continuous variable [75]. Fourth, there were concerns about recall bias and convenience sampling bias. In addition, habitual total sleep duration was self-assessed and was not objectively validated with polysomnography or actigraphy. Therefore, the results might reflect both overestimation or underestimation of the participants’ sleep duration [76]. Lastly, there was a potential sampling bias arising from exclusive online platform usage, volunteer bias due to self-selection of participants, the exclusion of a substantial number of non-respondents, and possible underrepresentation of linguistic minorities, all of which could impact the generalizability and validity of the findings.

## Conclusions

In this cross-sectional multi-national epidemiological study, we found that SARS-CoV-2 infection was associated with lower SRH, which was more pronounced in individuals with higher disease severity. In addition, lower SRH was associated with an increase in the number of post-COVID-19 symptom items. COVID-19 individuals who reported either decreased or increased habitual sleep duration following the infection also reported lower SRH. This study underscores the need to prioritize the consideration of habitual sleep duration for individuals infected with COVID-19 in public health initiatives; managing sleep may play a pivotal role in recovery. Further studies, preferably employing a prospective design utilizing clinical data, are needed to confirm the relation between changes in habitual sleep duration and health outcomes in individuals affected with COVID-19.

### Supplementary Information

**Additional file 1:** **Supplementary Table 1.** Exclusions.**Supplementary Table 2.** Multiple linear regression for self-rated health in participants who did not test positive for COVID-19 (*n* = 9,285). **Supplementary Table 3.** Multiple linear regression with country fixed effects for self-rated health in participants who did not test positive for COVID-19 (*n* = 9,285). **Supplementary Table 4.** Multiple linear regression for self-rated health in COVID-19 group (*n*= 1,509). **Supplementary Table 5.** Multiple linear regression with country fixed effects for self-rated health in COVID-19 group (*n*= 1,509). **Supplementary Table 6.** Ethical approval data.

## Data Availability

The datasets used and analyzed during the current study are available from the ICOSS second survey core group (FC, BH, IM, LDG, YKW, YD, and MP) on reasonable request.

## Abbreviations

BMI: Body mass index
COVID-19: Coronavirus disease 2019
CFS/ME: Chronic fatigue syndrome/myalgic encephalomyelitis
ICOSS- II: International COVID Sleep Study-II
OR: Odds ratio
SD: Standard deviation
SRH: Self-rated health
VAS: Visual analog scale
