# Supplementary material for: Associations between changes in habitual sleep duration and lower self-rated health among COVID-19 survivors: findings from a survey across 16 countries/regions
Source: BMC Public Health. 2023 Nov 28;23:2352. doi: 10.1186/s12889-023-17258-3 (PMC10683140; doi:10.1186/s12889-023-17258-3)
Supplement: Supplementary file 1 — Additional file 1: Supplementary Table 1. Exclusions.Supplementary Table 2. Multiple linear regression for self-rated health in participants who did not test positive for COVID-19 (n = 9,285). Supplementary Table 3. Multiple linear regression with country fixed effects for self-rated health in participants who did not test positive for COVID-19 (n = 9,285). Supplementary Table 4. Multiple linear regression for self-rated health in COVID-19 group (n= 1,509). Supplementary Table 5. Multiple linear regression with country fixed effects for self-rated health in COVID-19 group (n= 1,509). Supplementary Table 6. Ethical approval data. [file 12889_2023_17258_MOESM1_ESM.docx]

| **Supplementary Table 1**. Exclusions |  |
| --- | --- |
| Total number of survey responses to ICOSS-II | 15,813 |
| No report on self-rated health | 4,116 |
| Unclear country of origin | 44 |
| No report on male or female gender | 3 |
| No report on height or weight | 183 |
| No report on ethnicity | 66 |
| No report on marital status | 6 |
| No report on comorbid disorders | 11 |
| No report on financial burden | 7 |
| No report on vaccination information | 2 |
| Self-reported sleep duration <3h or >15h | 93 |
| No report on COVID-19 related questions | 488 |
| Final sample | 10,794 |

| **Supplementary Table 2.** Multiple linear regression for self-rated health in participants who did not test positive for COVID-19 (*n* = 9,285). | | | | |
| --- | --- | --- | --- | --- |
|  | B | Std. Error | *β* | *p* |
| Current habitual sleep duration |  |  |  |  |
| Short | −0.482 | 0.622 | −0.008 | 0.438 |
| Intermediate | (ref) |  |  |  |
| Long | **−10.044** | **1.499** | **−0.067** | **< 0.001** |
|  |  |  |  |  |
| Age | 0.000 | 0.012 | 0.000 | 0.988 |
| Gender: female (yes) | −0.412 | 0.389 | −0.011 | 0.290 |
| Body mass index | **−0.333** | **0.032** | **−0.105** | **< 0.001** |
| Ethnicity: Caucasian/white (yes) | **3.481** | **0.427** | **0.082** | **< 0.001** |
| Marital status: married/in relationship (yes) | **2.231** | **0.407** | **0.056** | **< 0.001** |
| Education: university, college or above (yes) | −0.529 | 0.415 | −0.013 | 0.202 |
| Number of comobidities^b^ | **−2.266** | **0.235** | **−0.103** | **< 0.001** |
| Number of COVID-19 vaccine doses^c^ | **0.951** | **0.266** | **0.036** | **< 0.001** |
| Financial burden^d^ | **−2.126** | **0.322** | **−0.067** | **< 0.001** |
| Sleep quality^e^ | **−5.376** | **0.179** | **−0.321** | **< 0.001** |
| ^a^Including hypertension, heart failure, stroke, diabetes, asthma, COPD, kidney failure, cancer, autoimmune disease, problems of movement and migraine. | | | | |
| ^b^Never=1, Once=2, Twice=3. | | | | |
| ^c^Not at all=1, A little/Somewhat=2, Much/Very much=3. | | | | |
| ^d^Well=1, rather well=2, neither well nor badly=3, rather badly=4, badly=5. | | | | |
| Analyses weighted by country representation and the joint age-gender distribution. | | | | |

| **Supplementary Table 3.** Multiple linear regression with country fixed effects for self-rated health in participants who did not test positive for COVID-19 (*n* = 9,285). | | | | |
| --- | --- | --- | --- | --- |
|  | B | Std. Error | *β* | *p* |
| Current sleep duration |  |  |  |  |
| < 6 h | **−1.501** | **0.575** | **−0.026** | **0.009** |
| 6 to 9 h | (ref) |  |  |  |
| > 9 h | **−7.006** | **0.945** | **−0.070** | **< 0.001** |
|  |  |  |  |  |
| Age | 0.027 | 0.014 | 0.023 | 0.051 |
| Gender: female (yes) | 0.217 | 0.423 | 0.005 | 0.608 |
| Body mass index | **−0.418** | **0.034** | **−0.126** | **< 0.001** |
| Ethnicity: Caucasian/white (yes) | −0.041 | 0.652 | −0.001 | 0.950 |
| Marital status: married/in relationship (yes) | **2.135** | **0.406** | **0.052** | **< 0.001** |
| Education: university, college or above (yes) | 0.396 | 0.416 | 0.009 | 0.341 |
| Number of comorbidities^a^ | **−3.499** | **0.256** | **−0.138** | **< 0.001** |
| Number of COVID-19 vaccine doses^b^ | 0.489 | 0.274 | 0.019 | 0.074 |
| Financial burden^c^ | **−3.275** | **0.311** | **−0.102** | **< 0.001** |
| Sleep quality^d^ | **−5.064** | **0.179** | **−0.288** | **< 0.001** |
| ^a^Including hypertension, heart failure, stroke, diabetes, asthma, COPD, kidney failure, cancer, autoimmune disease, problems of movement and migraine. | | | | |
| ^b^Never=1, Once=2, Twice=3. | | | | |
| ^c^Not at all=1, A little/Somewhat=2, Much/Very much=3. | | | | |
| ^d^Well=1, rather well=2, neither well nor badly=3, rather badly=4, badly=5. | | | | |
| Analyses are not weighted but control for country fixed effects. | | | | |

| **Supplementary Table 4.** Multiple linear regression for self-rated health in COVID-19 group (*n* = 1,509). | | | | |
| --- | --- | --- | --- | --- |
|  | B | Std. Error | *β* | *p* |
| Current habitual sleep duration |  |  |  |  |
| Short | −0.584 | 1.563 | −0.009 | 0.709 |
| Intermediate | (ref) |  |  |  |
| Long | −2.899 | 3.376 | −0.020 | 0.391 |
| Change in habitual sleep duration |  |  |  |  |
| ≤ −2 h | **−3.086** | **1.470** | **−0.051** | **0.036** |
| −2 to 1 h | (ref) |  |  |  |
| ≥ 1 h | **−4.491** | **1.531** | **−0.068** | **0.003** |
|  |  |  |  |  |
| Age | 0.017 | 0.032 | 0.013 | 0.596 |
| Gender: female (yes) | 1.478 | 0.977 | 0.034 | 0.131 |
| Body mass index | **−0.384** | **0.062** | **−0.139** | **< 0.001** |
| Ethnicity: Caucasian/white (yes) | **2.689** | **1.186** | **0.050** | **0.023** |
| Marital status: married/in relationship (yes) | 1.822 | 1.055 | 0.041 | 0.084 |
| Education: university, college or above (yes) | 0.179 | 1.038 | 0.004 | 0.863 |
| Number of comobidities^a^ | **−1.600** | **0.559** | **−0.072** | **0.004** |
| Number of COVID-19 vaccine doses^b^ | 0.911 | 0.586 | 0.034 | 0.120 |
| Financial burden^c^ | −0.689 | 0.728 | −0.022 | 0.344 |
| Severity of COVID-19^d^ | **−1.983** | **0.585** | **−0.079** | **< 0.001** |
| Number of post-COVID-19 symptoms^e^ | **−1.984** | **0.152** | **−0.345** | **< 0.001** |
| Sleep quality^g^ | **−3.268** | **0.449** | **−0.183** | **< 0.001** |
| ^a^Including hypertension, heart failure, stroke, diabetes, asthma, COPD, kidney failure, cancer, autoimmune disease, problems of movement and migraine. | | | | |
| ^b^Never=1, Once=2, Twice=3. | | | | |
| ^c^Not at all=1, A little/Somewhat=2, Much/Very much=3. | | | | |
| ^d^No marked symptoms =1, Mild=2, Moderate=3, Severe=4, Life threatening=5. | | | | |
| ^e^Including fatigue, brain fog/memory problems, postexertional malaise, joint/muscle pain, shortness of breath, loss of smell, sweating, headache, palpitations, tachycardia, dizziness, migraine, abnominal pains, feverishness, diarrhea, hypotension, urinary problems, and hallucinations. | | | | |
| ^f^Well=1, rather well=2, neither well nor badly=3, rather badly=4, badly=5. | | | | |
| Analyses weighted by country representation and the joint age-gender distribution. | | | | |

| **Supplementary Table 5.** Multiple linear regression with country fixed effects for self-rated health in COVID-19 group (*n* = 1,509). | | | | |
| --- | --- | --- | --- | --- |
|  | B | Std. Error | *β* | *p* |
| Current habitual sleep duration |  |  |  |  |
| < 6 h | −1.707 | 1.570 | −0.027 | 0.277 |
| 6 to 9 h | (ref) |  |  |  |
| > 9 h | **−5.240** | **2.266** | **−0.055** | **0.021** |
| Change in habitual sleep duration |  |  |  |  |
| ≤ −2 h | **−3.567** | **1.463** | **−0.059** | **0.015** |
| −2 to 1 h | (ref) |  |  |  |
| ≥ 1 h | **−4.345** | **1.563** | **−0.067** | **0.005** |
|  |  |  |  |  |
| Age | **0.054** | **0.037** | **0.110** | **< 0.001** |
| Gender: female (yes) | 0.434 | 0.946 | 0.033 | 0.126 |
| Body mass index | **−0.440** | **0.061** | **−0.171** | **< 0.001** |
| Ethnicity: Caucasian/white (yes) | −1.460 | 1.605 | −0.027 | 0.375 |
| Marital status: married/in relationship (yes) | **1.484** | **1.041** | **0.050** | **0.033** |
| Education: university, college or above (yes) | −0.969 | 1.058 | −0.035 | 0.125 |
| Number of comobidities^a^ | **−1.617** | **0.545** | **−0.110** | **< 0.001** |
| Number of COVID-19 vaccine doses^b^ | 0.066 | 0.602 | 0.029 | 0.198 |
| Financial burden^c^ | −2.057 | 0.712 | −0.038 | 0.092 |
| Severity of COVID-19^d^ | **−1.494** | **0.580** | **−0.060** | **0.009** |
| Number of post-COVID-19 symptoms^e^ | **−1.632** | **0.152** | **−0.333** | **< 0.001** |
| Sleep quality^f^ | **−3.198** | **0.446** | **−0.182** | **< 0.001** |
| ^a^Including hypertension, heart failure, stroke, diabetes, asthma, COPD, kidney failure, cancer, autoimmune disease, problems of movement and migraine. | | | | |
| ^b^Never=1, Once=2, Twice=3. | | | | |
| ^c^Not at all=1, A little/Somewhat=2, Much/Very much=3. | | | | |
| ^d^No marked symptoms =1, Mild=2, Moderate=3, Severe=4, Life threatening=5. | | | | |
| ^e^Including fatigue, brain fog/memory problems, postexertional malaise, joint/muscle pain, shortness of breath, loss of smell, sweating, headache, palpitations, tachycardia, dizziness, migraine, abnominal pains, feverishness, diarrhea, hypotension, urinary problems, and hallucinations. | | | | |
| ^f^Well=1, rather well=2, neither well nor badly=3, rather badly=4, badly=5. | | | | |
| Analyses are not weighted but control for country fixed effects. | | | | |

**Supplementary Table 6:** Ethical approval data.

| **Country** | **Ethical diary number** | **Notes** |
| --- | --- | --- |
| Austria | NA | Due to the anonymous nature of survey collection, the Ethical Board of the Medical University of Vienna, Austria did not require an ethical evaluation or approval (date: 2021-03-29). |
| Brazil | NA | Due to the anonymous nature of survey collection, the Ethics Committee of the Brain Institute - Federal University of Rio Grande do Norte did not require an ethical evaluation or approval (date: 2020-06-10). |
| Bulgaria | Protocol Number 46/05.08.2021 | Ethics Commission of the Institute of Neurobiology, Bulgarian Academy of Sciences |
| Canada | 2020-151-A-1-R-1 21-05-2021  REB#20-5540 | The “Comité d’éthique de l’Université Laval » reviewed and approved this research protocol.  University Health Network Research Ethics Board, Toronto, Ontario, Canada |
| China (Hong Kong) | 2020.277 | Joint Chinese University of Hong Kong-New Territories East Cluster Clinical Research Ethics Committee |
| Croatia | 100-21/21-4 (07.05.2021) | Ethics Committee of the Institute for Medical Research and Occupational Health |
| Finland | NA | Due to the anonymous nature of survey collection, the Finnish Institute for Health and Welfare did not require an ethical evaluation or approval (date: 2020-04-27). |
| France | NA | Due to the anonymous nature of survey collection, La Commission Nationale de l'Informatique et des Libertés did not require an ethical evaluation or approval (date: 2020-06-05). |
| Germany | EA1/162/20. | Ethics Committee of Charite University Hospital Berlin |
| Israel | AU-HEA-MK-20210603 | The study was approved by the Ariel University Human Research Ethics Committee of the Faculty of Health Sciences |
| Italy | protocol number: 0000861, April 24, 2021 | Institutional Ethics Committee of the Department of Psychology of the Sapienza University of Rome |
| Japan | No. 198/2020 | The ethics committee of the Neuropsychiatric Research Institute, Tokyo, Japan |
| Norway | NA | Due to the anonymous nature of survey collection, the regional komité for forskningsetikk, Vest Norge did not require an ethical evaluation or approval (date: 2020-05-20). |
| Portugal | CES-UCP nº142, May 27th, 2022 | The project was approved unanimously by the Life Sciences Ethical Commission of the Portuguese Catholic University. |
| Sweden | NA | Due to the anonymous nature of survey collection, the regional ethical board in Uppsala did not require an ethical evaluation or approval (date: 2020-06-25). |
| USA | IRB-20-257 | Deemed exempt by the Mississippi State University Institutional Review Board. |

*NA*, not applicable.

Informed consent was obtained where appropriate, and the procedures followed all principles stated by the Declaration of Helsinki.
